# Guidelines for the use of chest radiographs in community-acquired pneumonia in children and adolescents

**DOI:** 10.1007/s00247-017-3944-4

**Published:** 2017-09-21

**Authors:** Savvas Andronikou, Elena Lambert, Jarred Halton, Lucy Hilder, Iona Crumley, Mark D. Lyttle, Cara Kosack

**Affiliations:** 1 Department of Paediatric Radiology, Bristol Royal Hospital for Children, University of Bristol, Bristol, United Kingdom; 2 CRICBristol, 60 St. Michaels Hill, Bristol, BS2 8DX United Kingdom; 3 Diagnostic Network, Médecins Sans Frontières, Amsterdam, the Netherlands; 4 Emergency Department, Bristol Royal Hospital for Children, Bristol, United Kingdom; 5 Faculty of Health and Life Sciences, University of the West of England, Bristol, United Kingdom

**Keywords:** Children, Community-acquired pneumonia, Guidelines, Radiography, Ultrasound

## Abstract

National guidance from the United Kingdom and the United States on community-acquired pneumonia in children states that chest radiographs are not recommended routinely in uncomplicated cases. The main reason in the ambulatory setting is that there is no evidence of a substantial impact on clinical outcomes. However clinical practice and adherence to guidance is multifactorial and includes the clinical context (developed vs. developing world), the confidence of the attending physician, the changing incidence of complications (according to the success of immunisation programs), the availability of alternative imaging (and its relationship to perceived risks of radiation) and the reliability of the interpretation of imaging. In practice, chest radiographs are performed frequently for suspected pneumonia in children. Time pressures facing clinicians at the front line, difficulties in distinguishing which children require admission, restricted bed numbers for admissions, imaging-resource limitations, perceptions regarding risk from procedures, novel imaging modalities and the probability of other causes for the child’s presentation all need to be factored into a guideline. Other drivers that often weigh in, depending on the setting, include cost-effectiveness and the fear of litigation. Not all guidelines designed for the developed world can therefore be applied to the developing world, and practice guidelines require regular review in the context of new information. In addition, radiologists must improve radiographic diagnosis of pneumonia, reach consensus on the interpretive terminology that clarifies their confidence regarding the presence of pneumonia and act to replace one imaging technique with another whenever there is proof of improved accuracy or reliability.

## Introduction

Radiologists perceive that the role of imaging in community-acquired pneumonia is for the confirmation or exclusion of pneumonia, differentiation between viral and bacterial causes, exclusion of other possible causes for the symptoms and the detection of related complications. These are widely held perceptions despite evidence that radiographic findings are not useful for differentiating between viral and bacterial causation [1]. The role of imaging needs to be re-evaluated considering the increasing availability and safety of modalities such as ultrasound (US), computed tomography (CT) and magnetic resonance imaging (MRI) [2].

According to Bradley and colleagues [3], practice guidelines such as those for community-acquired pneumonia in children are “systematically developed statements to assist practitioners and patients in making decisions about appropriate health care for specific clinical circumstances”. In addition to representing validity, reliability, reproducibility, multidisciplinary process, review of evidence, and documentation, Bradley and colleagues stated that good guidelines must also have “clinical applicability, clinical flexibility [and] clarity” [3]. The updated (2011) guidelines of the British Thoracic Society for community-acquired pneumonia in children state that children with signs and symptoms of pneumonia who are not admitted to a hospital should not have a chest radiograph [4]. Despite these guidelines, chest radiographs are often performed for suspected pneumonia in children [5, 6]. In this paper, we discuss current perspectives on paediatric chest radiograph referral practice and radiographic findings in children with suspected community-acquired pneumonia in different clinical settings.

### Childhood community-acquired pneumonia in context

Globally there are 155 million cases of childhood pneumonia annually, and pneumonia results in the death of 2 million children younger than 5 years every year (20% of all deaths in this age group), making it the biggest single cause of childhood death [3]. In the United Kingdom the annual incidence of attendance to hospital for pneumonia is 14.4 per 10,000 in children ages 0–16 years, and 33.8 per 10,000 for those ages <5 years, with admission rates just slightly lower [4]. In the developing world, one South African study indicated a pneumonia incidence of 0.27 episodes per child-year and a case–fatality ratio of 1% for cases admitted to a hospital [7].

The definition of community-acquired pneumonia in children is almost identical in the United Kingdom and the United States. In the United States, it is “the presence of signs and symptoms of pneumonia in a previously healthy child caused by an infection that has been acquired outside of the hospital” [3]. According to the British Thoracic Society it is “the presence of signs and symptoms of pneumonia (such as fever of >38.5°C, cough and respiratory distress) in a previously healthy child, due to an infection which has been acquired outside the hospital” [8].

### Guidelines for imaging in suspected community-acquired pneumonia

National guidance on community-acquired pneumonia from the United Kingdom and the United States indicates that chest radiographs are not recommended routinely in uncomplicated cases [3, 4, 8]. However, there are defined situations where chest radiographs might be helpful in making the diagnosis [8], such as when there is pyrexia with only mild respiratory symptoms [3]. The main reason why chest radiography is not routinely recommended in the ambulatory setting is that there is no evidence of a substantial impact on clinical outcomes [3–5, 9, 10], which is also true for developing countries [3, 11]. This is because radiography cannot reliably differentiate viral from bacterial pneumonia and therefore does not impact on choice of treatment with antibiotics [4, 5].

Clinical practice and adherence to guidance is multifactorial and includes the clinical context (developed vs. developing world), the confidence of the attending physician, the changing incidence of complications (according to the success of immunisation programs), the availability of alternative imaging (and its relationship to perceived risks of radiation) and the reliability of the interpretation of imaging. The most recent British Thoracic Society guidance regarding community-acquired pneumonia is summarised in Table 1 [4] alongside the clinical practice guidelines by the Pediatric Infectious Diseases Society and the Infectious Diseases Society of America for the management of community-acquired pneumonia in infants and children older than 3 months [3]. It should be noted, however, that in creating these guidelines, when the evidence for the guidance is lacking, consensus is employed. In addition, guidelines are intended to be assistive, rather than directive, and therefore clinicians often operate outside the guidance in line with their clinical experience, e.g., it has been shown that junior doctors are more likely to use guidelines rigidly than more senior doctors, who tend to go on clinical intuition [12]. In practice, chest radiographs are performed frequently for suspected pneumonia in children [5]. An official British Thoracic Society audit identified that a chest radiograph had been performed to confirm the diagnosis in 90–94% of suspected community-acquired pneumonia cases and concluded that there was an over-reliance on investigations to diagnose pneumonia [6].

**Table 1:** Summary of the British Thoracic Society, Pediatric Infectious Diseases Society and the Infectious Diseases Society of America guidelines for the management of community-acquired pneumonia in infants and children

The British Thoracic Society guidelines [4] regarding chest radiography in the context of community-acquired pneumonia in children	Clinical practice guidelines by the Pediatric Infectious Diseases Society and the Infectious Diseases Society of America for the management of community-acquired pneumonia in infants and children older than 3 months of age [3]
Chest radiography is too insensitive to establish whether pneumonia is of viral or bacterial aetiology	Routine chest radiographs are not necessary for the confirmation of suspected community-acquired pneumonia in patients well enough to be treated in the outpatient setting
Chest radiography should not be considered a routine investigation in children thought to have community-acquired pneumonia	Chest radiographs, postero-anterior and lateral, should be obtained in patients with suspected or documented hypoxaemia or significant respiratory distress and in those with failed initial antibiotic therapy to verify the presence or absence of complications of pneumonia, including parapneumonic effusions, necrotizing pneumonia and pneumothorax
Children with signs and symptoms of pneumonia who are not admitted to a hospital should not have a chest radiograph	Chest radiographs (postero-anterior and lateral) should be obtained in all patients hospitalised for management of community-acquired pneumonia to document the presence, size, and character of parenchymal infiltrates and identify complications of pneumonia that may lead to interventions beyond antimicrobial agents and supportive medical therapy

Urbankowska and colleagues [13] claimed there are strong arguments in favor of performing diagnostic imaging in children with suspected community-acquired pneumonia. The main problem for clinicians is that even though the guidelines promote a clinical diagnosis, the signs and symptoms of lower respiratory tract infections are relatively nonspecific [13]. Under these conditions, the “need to prove the pneumonia” using some form of diagnostic imaging can be considered justified [13]. Even though many radiographs performed might not meet the British Thoracic Society guidelines for performing chest radiographs in children with suspected community-acquired pneumonia, these radiographs might also represent only a minority of all children seen in the emergency department for suspected respiratory infection. This performance is likely to arise from the alternative guidance around fever without source [14], which states that in some children in whom a source cannot be identified a chest radiograph should be performed if there are any respiratory symptoms. In this context the rationale may be to exclude pneumonia as a cause. In contrast to the guidelines, which only advocate use of imaging in severe pneumonia requiring hospitalisation, it is the mild cases presenting to the emergency department with a less-reliable history and examination that lead clinicians to request chest radiographs more commonly to support their diagnosis [9]. It was shown in one South African study that clinicians often mistakenly classify children with bronchiolitis episodes and those with reactive airway disease as having pneumonia [15]. The large number of equivocal cases seen in the emergency department has resulted in the so-called point-of-care US, which has been developed to enable a rapid diagnosis of lung consolidation and the complications of pneumonia, facilitating fast decision-making [16]. In the northern hemisphere, there is a marked seasonal pattern with winter preponderance for pneumococcal infection (December and January showing a peak 3–5 times higher than August) [4] and this might drive referrals.

The signs of community-acquired pneumonia might also be confusing. In the study by Zar and colleagues [7], 25% of chest radiographs for those admitted to hospital with pneumonia showed no radiologic signs of pneumonia. The inverse can also occur, in that radiographic infiltrates were reported on chest radiographs in 5–19% of children with fever in the absence of tachypnea, hypoxemia, respiratory distress or signs of lower respiratory tract infection. Chest radiography is recommended in this scenario, despite the absence of tachypnea or respiratory distress, because fever and cough might represent occult pneumonia [3]. However it is also possible that there is overcalling of pneumonia on radiographs, especially considering those reported as “radiographic infiltrates” rather than areas of “air–space consolidation”.

The reported incidence of empyema, complicating up to 7% of community-acquired pneumonia [6], also needs to be factored into the decision-making strategy because early detection is important for improved outcomes, especially in environments where the means for treating such complications are limited. Again, in this context, chest US might be indicated rather than chest radiography.

**Fig. 1:**
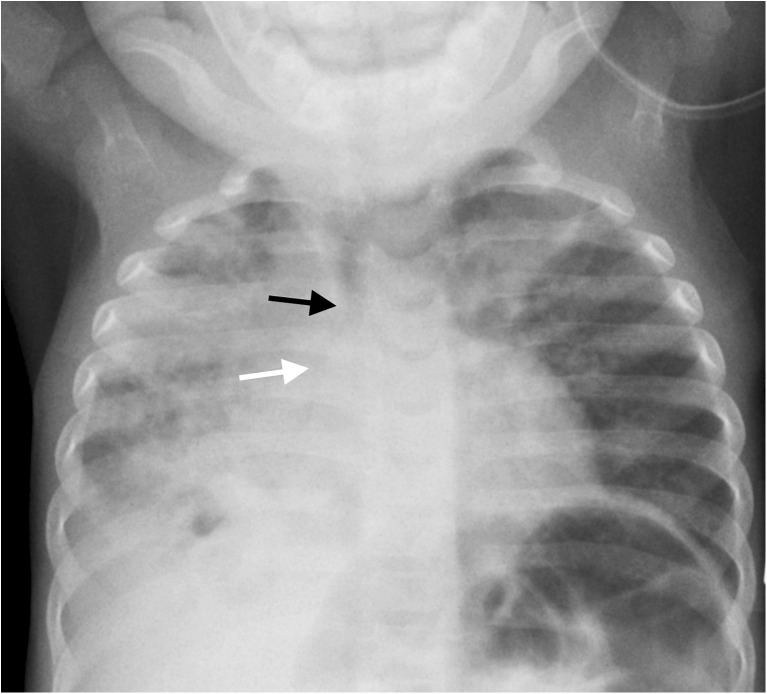
Anteroposterior chest radiograph requested in a malnourished 1-year-2-month-old boy presenting at a Médecins Sans Frontières site in the Central African Republic with an acute cough and lung crepitations. There was a specific request for the tele-reader to comment on any signs of pulmonary infection or signs of tuberculosis. The report read: “Infiltrates throughout the right lung and in the left upper lobe. Small cavity in the right lower lobe. Right hilar adenopathy narrows the right bronchus (*white arrow*) and mediastinal adenopathy slightly narrows the trachea (*black arrow*). Findings are highly indicative of primary TB”. Effusion on the right was not commented on

In contexts where tuberculosis is a consideration, the guidelines recommend a chest radiograph for a child with symptoms of community-acquired pneumonia with or without tuberculosis contact [17]. Chest radiographs form part of the diagnostic algorithm for tuberculosis, which can be undertaken at presentation or after failure to improve on antibiotic therapy [18]. In countries with a high tuberculosis prevalence, tuberculosis is cultured in only 8% of patients with community-acquired pneumonia but probably affects a larger number of these children as a co-pathogen [19]. In underserved settings, clinical signs are used for making a diagnosis of tuberculosis. In settings where radiologic investigations can be performed, the paucibacillary nature of tuberculosis in children has resulted in chest radiography remaining as one of the most utilised tools for making the diagnosis through the identification of mediastinal or hilar lymphadenopathy accompanying a parenchymal consolidation or focus [20] (Fig. 1).

### Diagnosis of pneumonia at chest radiography

**Fig. 2:**
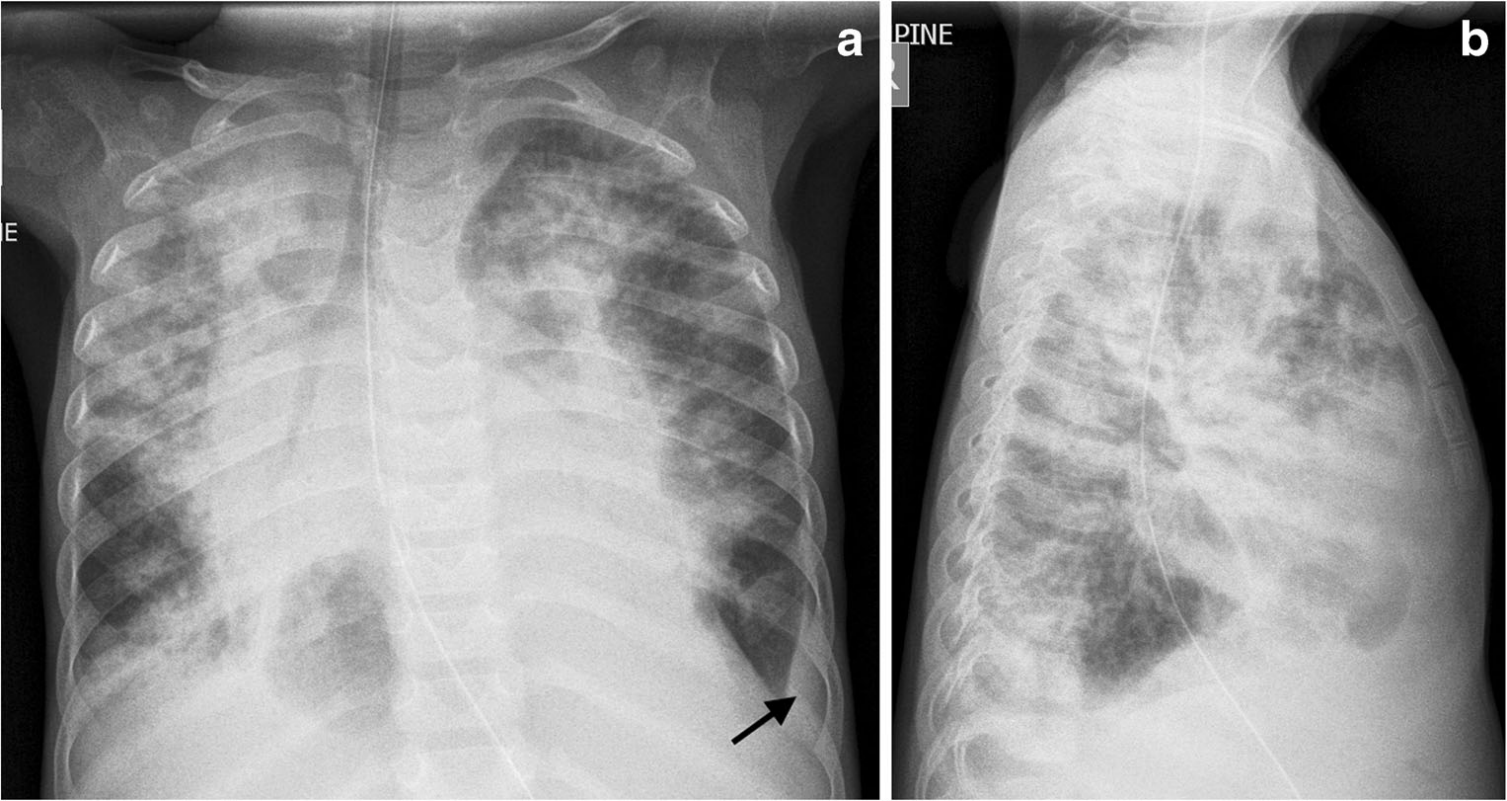
Imaging in a 6-year-old boy presenting with cough, dyspnoea, hepatosplenomegaly and oedema at a Médecins Sans Frontières site in the Democratic Republic of Congo. **a, b** Anteroposterior (**a**) and lateral (**b**) chest radiographs. The report read: “CXR shows widespread consolidation — there are many causes for multifocal pneumonia like this. TB is also possible as there is a small left basal pleural effusion (*black arrow*) and probable mediastinal adenopathy”. A round lucency in the right lower zone was reported as aerated lung and not a cavity

A prospective study in the United States showed pneumonia on chest radiographs in only 8.6% of patients referred for assessing this [4, 21]. A large study in Pakistani children with non-severe pneumonia reported a diagnosis of pneumonia on chest radiograph based on the World Health Organization (WHO) criteria in 14%, but only 1% of these constituted lobar consolidation, while the other 13% was made up of “interstitial parenchymal changes” [4, 22]. These two studies not only highlight different frequencies of radiographic findings in suspected pneumonia arising from different clinical settings, but also highlight how different terminology and interpretation of radiographs affect the frequency of disease recorded. In addition to the term “interstitial parenchymal changes” used to denote pneumonia, another contentious term is “infiltrates”, which is considered nonspecific and has been shown to have the highest discrepancy among readers [5, 23]. This term is often used in conjunction with the prefix perihilar (i.e. perihilar infiltrates) in the context of lung parenchymal infection. The lowest reported agreement between interpreters of chest radiographs is for what is described as “patchy and perihilar changes” [5], while agreement is better for “alveolar consolidation” [3]. It is therefore worth appraising publications regarding the proportion of chest radiographs reported as having consolidation/airspace disease (equivalent to alveolar consolidation) separately from those using vague terminology. There is also a higher reported reliability (interobserver agreement) for effusion than there is for consolidation [24] (Fig. 2).

Compounding the interpretation and descriptive differences of radiologists are the limitations of chest radiography itself. Interpretation of chest radiographs is subjective [3] and is challenging for both clinicians and expert radiologists [13]. This is because chest radiographs are two-dimensional and are therefore compromised by superimposition of normal and abnormal structures, with resultant summation artefacts and hidden pathology [8, 25]. The single-projection chest radiograph, as recommended by the British Thoracic Society guideline, masks up to 40% of lung by the overlying normal soft-tissue structures such as the heart and diaphragms [16]. Despite this, the current British Thoracic Society guidelines hold the position that a lateral view is unhelpful [16], while other groups such as the Pediatric Infectious Diseases Society, the Infectious Diseases Society of America [3] and the WHO [26] require anteroposterior and lateral views for diagnosis, as stated in their guidelines.

The subjective evaluation of radiographs and the differing interpretation of what is required from radiographs result in significant interobserver variability in the interpretation of these for community-acquired pneumonia [3, 5, 9, 16]. Radiologists may have a lower threshold for diagnosis, given their primary role is diagnosis, where they prefer to be inclusive rather than exclusive [27]. This can result in over-calling of pneumonia on chest radiographs. The WHO standardised criteria [4, 26] were developed with the goal to improve the interobserver agreement for the interpretation of radiographs in the context of paediatric pneumonia. Even though high agreement for identifying chest radiograph-confirmed pneumonia using these standardised definitions has been reported [3] the WHO definition of radiologic pneumonia has been criticised for resulting in overcalling of pneumonia and underestimating of the effect of pneumococcal conjugate vaccine [5, 28]. One significant area of contention is that the WHO criteria use the descriptive term “infiltrate”, which is not recommended in the Fleischner Society glossary of terms for thoracic imaging, because it has been used interchangeably for both pulmonary airspace and interstitial disease on both radiographs and CT scans and has meant different things to different people [29]. Chest radiography has also been shown to have a low sensitivity (compared to CT scans) [16], especially for small lung consolidations [30]. Therefore it is understood that the lack of any abnormality on chest radiography does not exclude community-acquired pneumonia [30] and that an abnormal chest radiograph might be interpreted as normal [5].

In addition to the concerns regarding the interpretation of chest radiographs in children, the feasibility and practicality of obtaining an adequate-quality chest radiograph in a timely fashion needs to be considered [16]. Quality assurance of chest radiographs is an ongoing activity in established and well-staffed Western institutions such as those in the United Kingdom, but has proved problematic in developing countries [31–33]. Chest radiography is therefore often considered impractical at the point of care [3] and has prompted the investigation of usefulness of lung US in the context of community-acquired pneumonia.

The perceived radiation risk from a single chest radiograph, which is one reason for discouraging its routine use, is a misrepresentation of current knowledge. Liszewski [34] reported that standard chest radiographs deliver a very low dose of ionising radiation of 0.01–0.02 mSv. The risk from such low doses is considered “almost non-existent” [35].

### In clinical practice

**Fig. 3:**
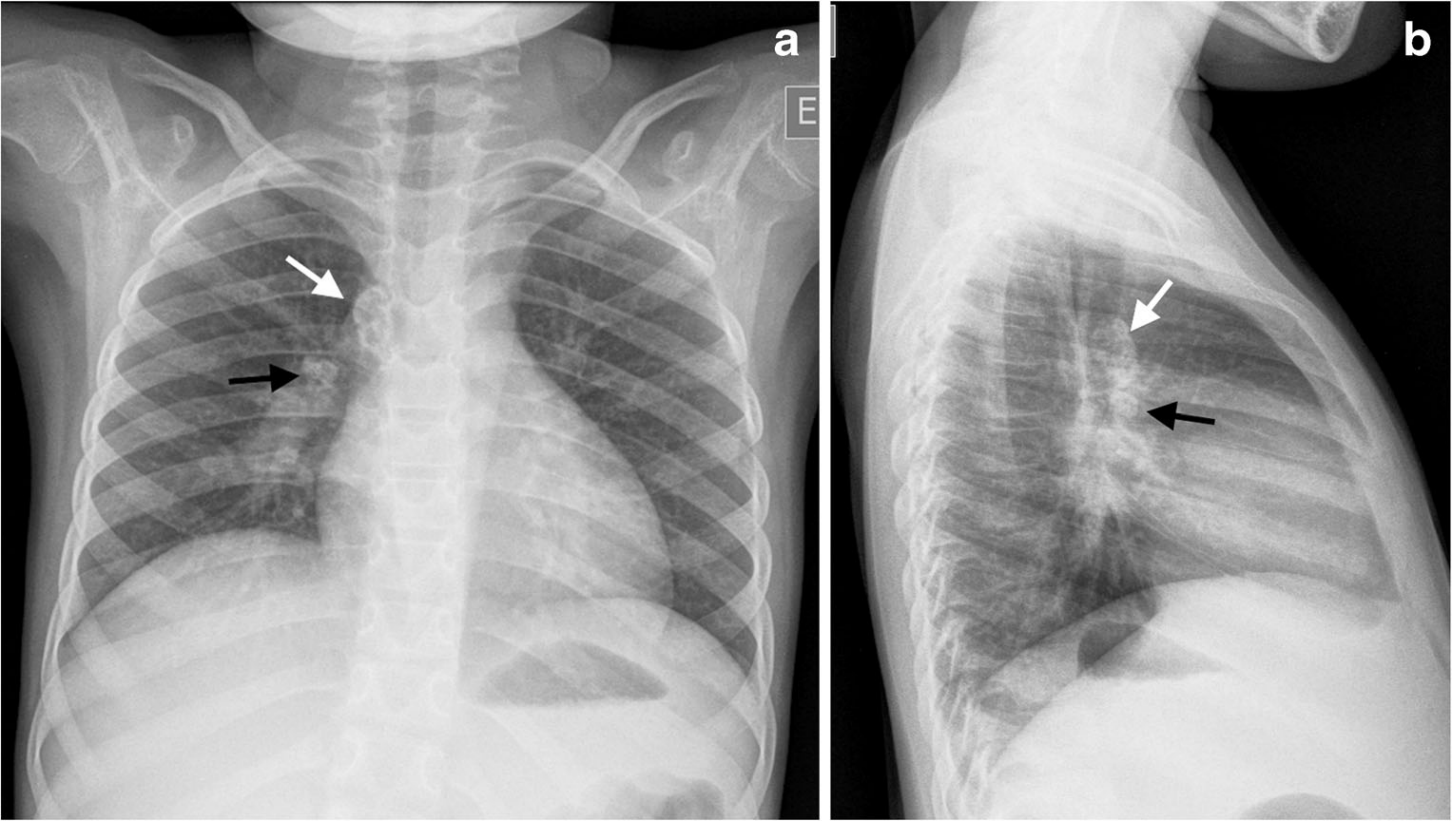
Imaging in an 8-year-old girl at a Médecins Sans Frontières site in the Democratic Republic of Congo referred for tele-reporting. Presenting symptoms were cough and fever, and the girl was not responsive to antibiotic treatment for suspected pneumonia. The request indicated that pneumonia and tuberculosis were being considered despite no known contact history. **a** Anteroposterior chest radiograph demonstrates calcified lymphadenopathy at the right paratracheal (*white arrow*) and right hilar regions (*black arrow*), consistent with primary pulmonary tuberculosis. **b** Lateral radiograph confirms the calcified paratracheal (*white arrow*) and hilar lymphadenopathy (*black arrow*)

It is important to remember that practice guidelines for community-acquired pneumonia in children are designed to assist practitioners in making decisions about appropriate care for specific clinical circumstances [3]. Harris et al. [4] made the distinction between the developed and developing world regarding the use of chest radiographs in this context, in that community-acquired pneumonia presenting in the developed world can be verified by the radiologic finding of consolidation while in the developing world there might be difficulties in obtaining a radiograph. However with the institution of more radiographic equipment in developing countries, guidelines need to address the practice-related benefits of this imaging modality rather than its availability [4] (Fig. 3). Time pressures facing clinicians at the front line, difficulties in distinguishing which children require admission, restricted bed numbers for admissions, imaging-resource limitations, perceptions regarding risk from procedures, novel imaging modalities and the probability of other causes for the child’s presentation all need to be factored into a guideline. More important, the these factors can be location-specific, related to a specific period and changed according to successful immunisation campaigns, and also might depend on the changing likelihood of complications. Other drivers that often weigh in to actual practice, depending on the setting, include cost-effectiveness and the fear of litigation. Not all guidelines designed for the developed world can therefore be applied to the developing world and practice guidelines require regular review in the context of new information.

## Conclusion

There is ongoing use of chest radiographs in the management of suspected community-acquired pneumonia in children by clinicians in the developed world that runs contrary to the recommendations of existing guidelines. There is also continued use of chest radiographs by clinicians in the developing world who have a frequent and well-founded concern regarding the identification of tuberculosis as a cause of acute respiratory disease. Guidelines are designed to assist clinicians but should not be considered prescriptive, especially considering the rapidly evolving presentation of pneumonia, ongoing advances in imaging technology, changes in patient management and differing geographic contexts of the practitioner. In addition, radiologists must work to improve radiographic diagnosis of pneumonia, reach consensus on the interpretive terminology that clarifies their confidence regarding the presence of pneumonia and act to replace one imaging technique with another whenever there is proof of improved accuracy or reliability.
